# From Methodological Challenges to Recommendations for Future Practice: Lessons From an Ethnographic Study With People With Intellectual Disabilities

**DOI:** 10.1111/jar.70266

**Published:** 2026-06-21

**Authors:** Karina Nissen Frøkjær, Siri Lygum Voldbjerg, Tine Fristrup, Philippa Rasmussen, Mette Grønkjær, Helle Haslund‐Thomsen

**Affiliations:** ^1^ Department of Clinical Medicine Aalborg University Aalborg Denmark; ^2^ Clinical Nursing Research Unit Aalborg University Hospital Aalborg Denmark; ^3^ Department of Nursing University College of Northern Denmark Aalborg Denmark; ^4^ Danish School of Education Aarhus University Aarhus Denmark; ^5^ Adelaide Nursing School University of Adelaide Adelaide South Australia Australia

**Keywords:** inclusive research, intellectual disability, interviews, methodology, observations, qualitative research

## Abstract

**Background:**

Methodological, social and ethical challenges embedded in qualitative research with people with intellectual disabilities call for innovation and approaches that amplify their voices. This paper aims to offer methodological recommendations based on a critical reflection and discussion of the challenges involved in conducting qualitative and inclusive research with people with intellectual disabilities. It focuses on challenges related to inclusion, obtaining consent, conducting participant observations and individual interviews and involvement through an expert panel including people with intellectual disabilities.

**Method:**

Inclusive research and critical disability studies inspired the analytical lens applied in the discussion.

**Results:**

Researchers are advised to adapt and tailor research methods. This may involve adopting an inclusive approach, establishing prior familiarity, applying multimodal strategies or alternative methods to verbally dominated approaches, such as the sensory‐dialogical approach.

**Conclusion:**

This paper contributes to existing research methodologies; however, substantial challenges remain and future methodological developments are needed.

## Lay Summary

- Use Easy Read and plain language when asking for consent, so it's clear and simple. Verify consent regularly, rather than asking just once.
- When taking notes during observations, respect what participants want and need.
- Before interviews, get to know the person and use that knowledge to make communication easier.
- If people with intellectual disabilities are part of an expert panel, make sure everyone has an equal chance to take part.

## 1 Introduction

Ethnography is a widely recognised qualitative research methodology characterised by its flexible approach and by the researcher's prolonged engagement in the everyday lives of participants across different populations and cultures. Ethnographic studies draw on data generated from multiple sources, with participant observation and informal interviews serving as the primary modes of data collection (Hammersley and Atkinson 2019; Reeves et al. 2008). Conducting qualitative research with people with intellectual disabilities seeks to ensure that studies are firmly grounded in the lived experiences and perspectives of participants (Beail and Williams 2014; Nind 2008). In the past, people with intellectual disabilities have been significantly underrepresented and frequently excluded from research (Beail and Williams 2014; Coons and Watson 2013; McDonald et al. 2021). Their perspectives have historically been marginalised by systematic barriers embedded in conventional research paradigms with emphasis placed on cognitive and communicative limitations, factors that influence comprehension, informed consent capacity and expressive ability (McFarland et al. 2024). However, adaptive approaches that critically engage with the problematisation of research methods, aiming to tailor methodologies to enable the inclusive participation of people with intellectual disabilities, have begun to gain traction (Gjermestad et al. 2023; Hollomotz 2017; McFarland et al. 2024). However, there are still methodological, ethical and social concerns that create barriers to their participation and increase the likelihood of their exclusion in research (Gjermestad et al. 2023; McDonald et al. 2017, 2021; McFarland et al. 2024). This is of concern, given that recent scientific advances hold considerable promise for quality of life improvements for this population. Realising this potential necessitates the active and meaningful involvement of people with intellectual disabilities throughout the research process (McDonald et al. 2017). Furthermore, people with intellectual disabilities are often highly motivated to engage in research (McDonald et al. 2021), and their exclusion may be experienced as harmful (McDonald et al. 2017).

Even though people with intellectual disabilities may face challenges in articulating their feelings and internal emotional states, employing methodologies that maximise opportunities for self‐expression can significantly enhance the quality of the data collected while reducing reliance on observable behavioural indicators (Prosser and Bromley 2012). This calls for further exploration of methodological challenges and the development of appropriate ways to enable greater participation and amplify the voices of people with intellectual disabilities in various aspects of the research process (Beail and Williams 2014; Nind and Vinha 2014; Walmsley et al. 2018). This is central to the inclusive research approach, which is grounded in emancipatory values and requires researchers to design methodologies that uphold dignity, ensure the perspectives of people with intellectual disabilities are heard, and enable meaningful influence over research processes (McFarland et al. 2024; Milner and Frawley 2018; Nind and Vinha 2014; O'Brien et al. 2022). Although such involvement may present challenges, it also offers important opportunities that can enrich both the research process and the knowledge it produces (Nind and Vinha 2014; Walmsley et al. 2018).

To address the knowledge gaps in this area, this paper builds on the authors' experiences from conducting an ethnographic study aimed at exploring factors shaping the decision‐making process related to health among people with intellectual disabilities living in group homes. Throughout the study, several methodological reflections and decisions were undertaken to respect, capture and reflect the lived experiences of people with intellectual disabilities. Some of these reflections are presented in this paper by discussing challenges with inclusion, obtaining consent and collecting data through participant observations and individual interviews.

Furthermore, the paper presents reflections on inclusive research informed by the experiences of involving people with intellectual disabilities in an expert panel. While the study is positioned within inclusive research, critical reflection reveals how our methodological choices inadvertently reproduced what Goodley et al. (2025) identify as ableist assumptions embedded within contemporary research paradigms.

The aim of this paper is to offer methodological recommendations based on a critical reflection and discussion of the challenges involved in conducting qualitative and inclusive research with people with intellectual disabilities. This contributes to the expanding body of scholarship that examines not only how to include people with intellectual disabilities in research but also how intellectual disability challenges fundamental epistemological assumptions about knowledge production, human autonomy and scholarly authority (Nind 2017; Srdanovic et al. 2024).

## 2 Methods

This section defines and contextualises the inclusive research and critical disability studies that have inspired the analytical lens applied in the subsequent critical reflection and discussion. Moreover, it outlines the ethnographic study that forms the basis for the discussion.

### 2.1 Defining and Contextualising Inclusive Research and Critical Disability Studies

Inclusive research offers a wide range of potential modes of involvement for people with intellectual disabilities, many of which are informed by participatory, action‐oriented or emancipatory research designs and methodologies (McFarland et al. 2024; Walmsley and Johnson 2003). To support an inclusive research approach, this study established an expert panel. The aim was to capture and reflect the members' lived experiences, respecting their diverse perspectives, incorporating their contribution in decisions throughout the research process, and ensuring that the study's findings would be relevant and beneficial to the broader population of people with intellectual disabilities (Nind 2017; O'Brien et al. 2022; Walmsley and Johnson 2003).

Inclusive research represents a fundamental shift in disability studies from traditional research paradigms that position disabled people as passive subjects to approaches that centre their voices, experiences and agency as active participants and co‐researchers (Walmsley and Johnson 2003). This methodological approach reflects broader movements within disability studies that challenge ableist assumptions and seek to redistribute power within the research process (Goodley and Runswick‐Cole 2014). As articulated by Nind (2014), inclusive research encompasses approaches where research questions are owned by or have relevance to people with intellectual disabilities, further their interests and ensure collaboration and accessibility throughout the research process. The theoretical underpinnings of inclusive research draw heavily on critical disability studies, which position disability not as an individual deficit but as a social and political construct that reveals the limitations of normative research methodologies (Goodley et al. 2021). This perspective aligns with what Goodley and Runswick‐Cole (2014) describe as ‘dishuman’ methodological approaches, which simultaneously acknowledge people with intellectual disabilities' humanity while recognising how disability challenges traditional humanist notions of autonomous, rational subjectivity. Disability scholars describe inclusive research as a progression from research ‘on’ disabled people to research ‘for’, ‘with’ and ultimately ‘by’ them (Frankena et al. 2015). This progression reflects broader shifts within disability studies towards recognising people with intellectual disabilities as knowledge producers rather than merely subjects of inquiry. However, as Gjermestad et al. (2023) argued, even well‐intentioned inclusive research often operates within criteria that exclude people with profound and multiple learning disabilities, highlighting the need for continued methodological innovation.

### 2.2 Ethnographic Study

The experiences discussed in this paper derive from an ethnographic study aimed at exploring factors shaping the decision‐making process related to health, among people with intellectual disabilities living in group homes. Participants included 11 female and 14 male adults with intellectual disabilities (*n* = 25), aged 23–59 (median 41) living with mild to severe intellectual disability. The ethnographic fieldwork took place over a 10‐month period from June 2022 through March 2023 at three municipal group homes in a suburban area near a larger city in Denmark. The data collection consisted of 434 h of participant observations, supplemented by informal interviews and semi‐structured individual interviews with six female and nine male participants (*n* = 15) with mild to severe intellectual disabilities aged 23–59 (median 39). The first author collected all the data and facilitated an expert panel. The expert panel was established at the outset of the research project and comprised two people with intellectual disabilities (one male and one female), both serving as chairpersons of the Danish disability organisation ULF. Also represented were a project and activity manager from ULF, the national chairperson of LEV, an interest organisation for people with intellectual disabilities, a support worker and a family member of a person with an intellectual disability. The involvement of people with intellectual disabilities and collaboration with relevant organisations is important to ensure the results are useful for the broader population (Walmsley et al. 2018). The members participated in six mixed online and physical dialogue‐based meetings discussing different phases of the research process, including ethical considerations, obtaining consent, entry and exit from the field, development and pilot test of the interview guide, and validation of the results.

In the following, challenges experienced conducting an ethnographic study with people with intellectual disabilities will be critically reflected on and discussed. The discussion is divided into the following sections: (1) Including people with intellectual disabilities and obtaining their consent, (2) navigating field role in participant observation, (3) emotional safeguarding and flexible, tailored communication in qualitative interviews and (4) balancing power dynamics when involving people with intellectual disabilities and other relevant experts.

## 3 Including People With Intellectual Disabilities and Obtaining Their Consent

The most crucial ethical principle in human studies is ensuring that individuals can consent to participate with a full understanding of what will occur (Emerson et al. 2004). This principle was challenged during the inclusion process in this study, as it was necessary to balance protecting individuals from coercion or participation without genuine consent while also ensuring they were not unnecessarily excluded, especially when the research necessitated participation across multiple ability levels (Cameron and Murphy 2006). Moreover, participants needed additional time to reflect before giving consent, and a formal informed consent form that was not tailored to the needs of people with intellectual disabilities further challenged the process of obtaining informed consent.

### 3.1 Balancing the Protection and Inclusion of People With Intellectual Disabilities in Research

A dilemma arose in determining who to invite to participate in this study: there was both a need to protect individuals from coercion or participation without genuine consent and an imperative to avoid unnecessary exclusion. This dilemma reflects the tension within policy frameworks in Western’ countries, which portray people with intellectual disabilities as autonomous agents while simultaneously emphasising the need to protect them as vulnerable adults (Banks Carys 2018). Consent‐based vulnerabilities are more pronounced in people with intellectual disabilities, as some may lack the capacity to provide fully informed consent or struggle to express their autonomy (Kiyimba et al. 2019). Others might be able to provide consent (with support), give assent or express signs of dissent. Consequently, people with intellectual disabilities have a reduced ability to safeguard their own interests and are more likely to bear the burdens of participation, requiring additional protection to ensure their well‐being, which has sometimes resulted in their exclusion (Kiyimba et al. 2019). Cameron and Murphy (2006) found that participants with intellectual disabilities who had lower comprehension levels struggled to understand their research study involvement. While they may grasp the essence of the research project, they may not fully comprehend the associated risks and benefits or their right to decline participation or withdraw from the study (Emerson et al. 2004). Including participants based on degree of disability can be unreliable and disrespectful, as inclusion should rather be based on potential participants ability to express an adequate preference and interest in engaging in research (Cameron and Murphy 2006; McDonald et al. 2012). Emerson et al. (2004) stated that a minimum requirement for consent is the ability to express whether the person wants to participate. However, studies point out that the disability should not automatically result in the inability to provide informed consent to participate in research (Horner‐Johnson and Bailey 2013; McDonald and Kidney 2012). Despite this, no universal guidelines exist for determining the cognitive capacity to consent to participate in research (Emerson et al. 2004). The absence of guidelines created tension regarding who to invite to participate in this study. Ultimately, we invited participants to participate on a case‐by‐case basis, assessing each potential participant's capacity to provide informed consent (Lacono 2009). The assessment required that potential participants be able to clearly express whether they wished to participate in the research. This included people with mild to severe intellectual disabilities, all of whom could sufficiently express themselves verbally, although some had limited verbal language and supplemented their speech with nonverbal communication.

**FIGURE 1:**
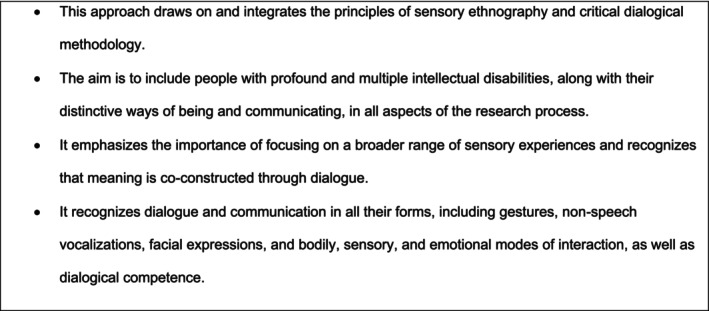
Central elements from the sensory‐dialogical approach, adapted from Gjermestad et al. (2023, 41, 50).

An alternative way to include participants could have been to use the approach by Gjermestad et al. (2023), who argued that a productive way to rethink inclusive research is by adopting a sensory‐dialogical approach, offering a promising pathway for including this group, and thereby acknowledging them as citizens (see Figure 1). Had the sensory‐dialogical approach been applied in our study, it could have enabled the inclusion of people with profound intellectual disabilities, as this approach acknowledges their unique ways of being and communicating. This perspective thus challenges traditional notions of communication and participation by expanding what counts as meaningful interaction in research involving this population (Gjermestad et al. 2023).

### 3.2 The Necessity of Adequate Time to Understand and Decide on Participation

To collect and use the information provided by participants, informed consent is required. In this study, we faced a significant challenge because consent could not always be immediately given. Potential participants often needed time to understand the researcher's role and feel comfortable participating. This meant that sensitive information could not be used until consent was formally obtained, nor could the first author who collected the data prevent this information from being retained in memory, forcing the researcher to responsibly balance ethical and legal considerations. Other studies support that people with intellectual disabilities require more time to understand the research and make decisions about participation (Banks Carys 2018; Cameron and Murphy 2006). It may be necessary to repeat information or break it down over an extended period of time (Cameron and Murphy 2006; Emerson et al. 2004). In this study, several strategies were implemented to clarify the researcher's role, provide information about the study, and foster both trust and comfort regarding participation.

The first author spent 2–4 days at each group home prior to obtaining informed consent. These visits included time spent in the common areas, engaging in informal conversations and answering questions about the project. Potential participants were provided with an oral presentation in plain language, supported by a PowerPoint with details about the project, participation opportunity, with time for dialogue and questions. In addition, a poster was displayed in each group home prior to data collection, serving as an information letter. The poster featured a photo of the first author who collected the data, an illustration of a book to represent the outcome, and brief text in simple language explaining the study purpose, the process of data collection and the participation opportunity (see Appendix A). Since it turned out that providing information extended over a longer period, this process included a temporal dimension and a progression in the fieldwork, during which the first author's presence became increasingly familiar to the participants. Based on this, the first author adopted a pragmatic approach by initially observing participants who were comfortable giving informed consent. As additional consents were obtained, data collection progressively expanded to include a larger group of participants.

### 3.3 Tailoring Formal Informed Consent

Securing formal informed consent proved to be a complex and challenging aspect of the research process in this study, as not all the participants fitted into the conventional notion of an ‘autonomous individual’ (Banks Carys 2018). Therefore, it is essential for the researcher to ensure that potential participants fully comprehend the provided information by using available support and tailoring the communication to the specific needs and capacity of those whose participation is being sought (Emerson et al. 2004; Harding 2021).

The expert panel helped adapt and tailor the formal informed consent process. Their knowledge and experience contributed to adapting the procedure with a focus on balancing information delivery, using tailored communication supported by illustrations, highlighting the right to refuse participation and involving a support person when necessary (Cameron and Murphy 2006; Emerson et al. 2004). This is consistent with previous research emphasising the crucial role of effective communication (Cameron and Murphy 2006; Emerson et al. 2004; Newcombe 2022). The Easy Read format is designed to make information more accessible and better aligned with individual preferences (Newcombe 2022). Moreover, researchers should present the core elements of the study in clear and accessible language, avoiding technical jargon and unnecessary complexity. They should also supplement verbal explanations with multimodal strategies, such as visual aids to clarify the research purpose and procedures, thereby reducing abstraction (Cameron and Murphy 2006; Emerson et al. 2004).

Obtaining consent took place as a dialogue between the first author and the potential participant, accompanied by a support worker. Involving a support worker was intended both to create a safe and trusting environment based on their existing relationship and to assist the first author in communicating in a way tailored to the potential participants' comprehension (Cameron and Murphy 2006; McDonald et al. 2012; Newcombe 2022). Emerson et al. (2004) emphasised the importance of a trusted support person if the person with intellectual disabilities tends to be acquiescent or compliant or has limited experience in decision‐making. Socially disadvantaged people with intellectual disabilities may welcome a researcher's intrusion, or the researcher may simply be another figure in a long line of workers passing through their lives. These issues are challenging to identify in advance, unless the researcher is already familiar with the potential participants. Therefore, a potential participant might need a trusted support person or advocate to help them understand the information and decide whether to participate (Emerson et al. 2004). Even though a trusted person might be helpful, researchers must be aware of the complexity of consent contexts. If the person from whom consent is sought tends to rely on others in decision‐making, it suggests a susceptibility to the views of those around them. These dynamics are deeply ingrained in the institutional context of care provider and care receiver relationships within social care settings and have significant implications for formal ethical codes, such as the processes of informed consent (Banks Carys 2018).

Despite various efforts to tailor the consent process, it cannot be guaranteed that participants have fully understood the information provided, nor can it be excluded that their decision has been influenced by others' opinions (Banks Carys 2018; Emerson et al. 2004). Maes et al. (2021) highlighted the importance of continuously monitoring participants' willingness to engage in a given study throughout the entire data collection process. Therefore, in addition to formal informed consent, this study used process consent (Banks Carys 2018; Harding 2021). This approach seeks ongoing consent rather than relying on one‐time formal consent. Process consent recognises that participation preferences might change over time and may be a strategy to increase certainty about the participants' sincere desire to participate (Banks Carys 2018). In our study, the process consent was based on both verbal and non‐verbal indications of the participants' preferences to engage on any given day, fostering a flexible and modular approach to participation.

An additional consideration pertains to the principle that when a person is considered unable to provide informed consent, a substitute decision‐maker (proxy) must make the decision on their behalf, although the legal requirements for this process vary across jurisdictions (Emerson et al. 2004; Newcombe 2022). In Denmark, only a guardian responsible for personal matters can provide such consent. In our study, one of the participants had a personal guardian. Legally, the guardian's consent was required; however, it was deemed essential to respect participants' autonomy, integrity and rights and ensure that they genuinely wished to take part (Holmqvist et al. 2022). Therefore, the process of ongoing assent also constituted a mandatory component in this case.

## 4 Navigating the Field Role in Participant Observation

The first author's involvement in the daily lives of people with intellectual disabilities inevitably influenced the research setting (Hammersley and Atkinson 2019). As social research cannot be conducted in isolation from society or personal characteristics, the researcher must be reflexive about the impact of their presence in the field (Atkinson 2015; Hammersley and Atkinson 2019). In this study, the first author faced challenges in openly taking field notes and, moreover, in managing the process of exiting the field and the potential harm it might cause to emotionally attached participants. The reflexive practice included the participants' reactions to field notes taken openly. Some participants expressed distraction through their body language or verbally expressed concern about what may have been written down about them. These reactions were addressed by amending the approach to field note documentation. In situations where participants became distracted, the first author relied on ‘head notes’ rather than written ones, allowing for a more flexible and context‐sensitive method to capture data (Emerson et al. 2011). If the participants expressed a concern, the first author asked for permission to write down what had just been said, contributing to transparency and a trusting atmosphere around taking field notes. These experiences with taking field notes highlight the emergent need for situated and adaptive note‐taking practices, which became essential to ensure alignment with the situation, individual preferences and the specific context.

Reflexive practice also involved addressing challenges related to participants' emotional attachments. During the participant observations, the first author became closely involved in the participants' daily lives. Such close involvement was also experienced in the ethnographic study conducted by Banks Carys (2018) where people with learning disabilities became emotionally attached to the researcher during the relationship‐building process, raising concerns about the potential harm of intermittently entering and exiting the participants' lives, particularly for those who might experience loneliness. On this basis, we ensured that the purpose and limited duration of the first author's presence were clearly communicated from the outset, supported by a poster for clarity (see Appendix A). Moreover, to avoid confusion about the role of the researcher, the first author adapted the field role and refrained from engaging in tasks such as emotional support or support for structured daily routines or activities, such as dressing and toileting. We found that this up‐front clarity and positioning in the field may have helped participants manage the entry and exit from the field.

## 5 Emotional Safeguarding and Flexible, Tailored Communication in Qualitative Interviews

Interviewing people with intellectual disabilities allows for a deeper understanding of their subjective experiences, attitudes and beliefs (Prosser and Bromley 2012). However, this study faced challenges related to participants' concerns about the interview process potentially leading to nervousness or anxiety. Moreover, the participants' unique ways of understanding and expressing themselves challenged both the traditional, verbally dominated interview techniques and the first author's own communicative competencies.

### 5.1 Addressing and Managing Potential Nervousness or Anxiety

When the participants were introduced to the possibility of participating in an individual interview, some expressed uncertainty and concern, particularly due to their lack of prior experience with interviews. Therefore, it was crucial to proactively address and mitigate this challenge, as such uncertainty can present as nervousness or anxiety during interviews. Creating a safe and supportive environment encourages greater engagement among participants with communication impairments (Emerson et al. 2004).

**FIGURE 2:**
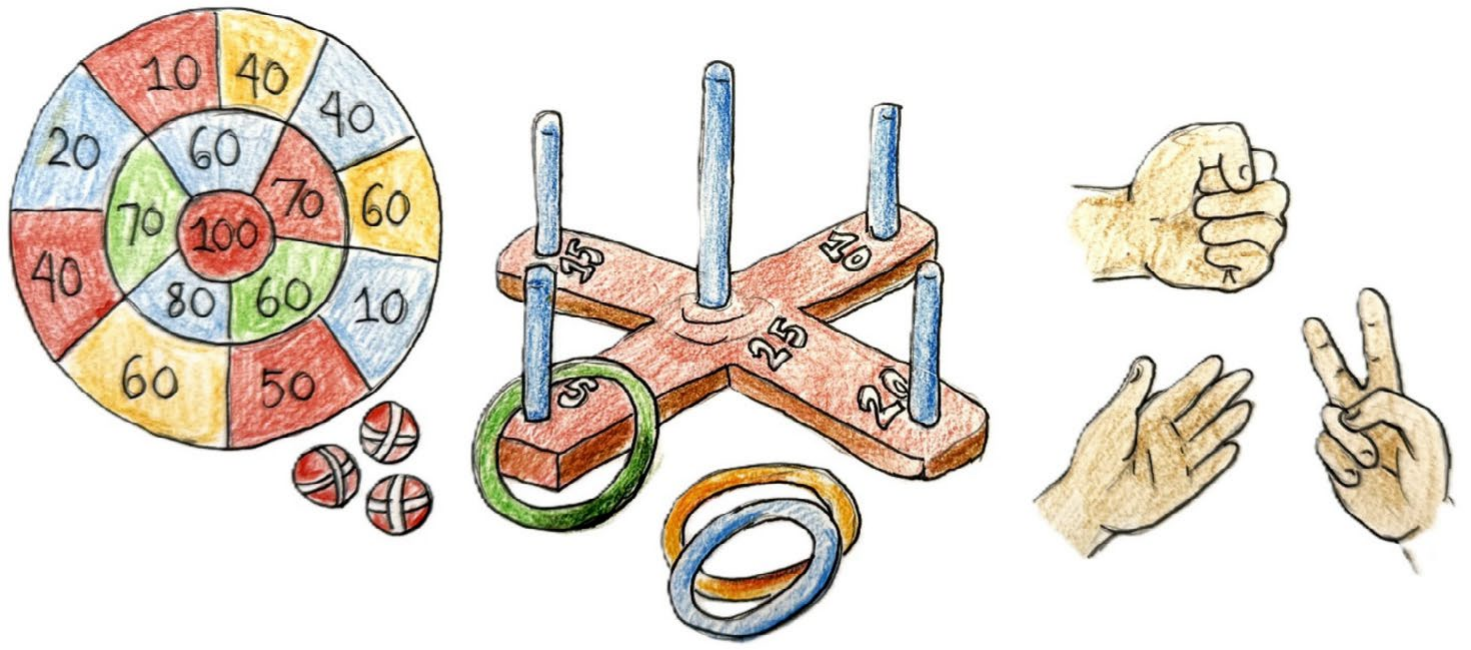
The illustrations from which the participants could choose a game.

Emerson et al. (2004) stated that prior to conducting interviews, the researcher must anticipate any potential anxiety the interview may cause and develop a strategy to manage it effectively. If it remains after the interview, the researcher should have some support arranged. Therefore, minimising potential nervousness or anxiety involved maintaining attentiveness throughout the interview process before, during and after. Before conducting the interviews, the participant was encouraged to choose the setting. These settings consisted of a familiar undisturbed location, such as the participants' apartment or a meeting room, which may have contributed to a more relaxed atmosphere (Prosser and Bromley 2012). Regardless of whether a person has an intellectual disability, there's often some initial nervousness. Therefore, it is generally not advisable to go straight into asking questions for the study (Emerson et al. 2004). Inspired by Emerson et al. (2004), this study started with an ‘informal chat’ about a neutral topic to help ease the potential nervousness or anxiety. To bolster the participants' confidence, the first author stated that there could be no ‘wrong’ answers. Previous research has found that prior knowledge of the participants' ways of communicating can be useful when conducting interviews (Hollomotz 2017). Accordingly, the first author became familiar with the participants through prior participant observations. This insight included understanding their interest in competition, which was used to foster a relaxed atmosphere before the formal interview. Depending on the participants' preferences, some were invited to play a game with the first author (see Figure 2), which triggered the giving of a gift, such as a mug, socks or a hair clip. Others who were averse to competition were not offered a game but instead received a gift at the end of the interview to acknowledge their contribution.

During the interviews, some people with learning, communication or other disabilities might benefit from the emotional support of a trusted and familiar person (Nind 2008). However, in our study, participants did not express preferences to have a trusted third party present, indicating a trusted relationship with the first author had already been established through the preceding months of participant observations. Had this prior relationship‐building not taken place, it is conceivable that a trusted third party would have been necessary. After the interviews, the first author ensured that support workers were always aware of the interviews and available. At the end of each interview, the participants were asked about their interview experiences. Subsequently, the support workers were briefed about the participants' experience and the first author's' own perception of the participant's emotional state during and at the end of the interview to provide the best possible post‐interview support, if needed. This study found that prior participant observations informed the methodological approach to managing potential nervousness or anxiety. Moreover, the attention given before, during and after the interview helped prevent significant nervousness or anxiety that might otherwise have been exacerbated in the absence of such initiatives.

### 5.2 Flexible, Tailored Communication in Qualitative Interviews

Based on knowledge gained about the participants prior to the formal individual interviews, this study recognised the importance of continuously tailoring communication to each participant's unique way of understanding and expressing themselves. This both challenged the first author's own communicative competencies, due to a lack of prior experience with interviewing this target group, and the traditional, verbally dominated interview techniques (Gjermestad et al. 2023).

Given the first author's lack of prior experience, it was deemed essential to involve the expert panel in developing the interview guide and crafting relevant, tailored interview questions. This aligns with what Erevelles (2011) terms a ‘materialist disability studies’ approach, which connects methodological innovation to broader struggles for social justice. This means ensuring that research practices not only include people with intellectual disabilities but also actively contribute to challenging the systems that marginalise them. It requires what Charlton (1998, 3) famously articulated as ‘nothing about us, without us’; not merely consultation but genuine power‐sharing in determining research priorities, methods and applications.

The expert panel emphasised several key points, including the importance of speaking slowly, avoiding jargon, incorporating body language, and utilising pictures, drawings and photos for support. Moreover, they emphasised the value of different versions of interview questions to better accommodate participants' needs. This resulted in the development of a semi‐structured interview guide with primarily open‐ended questions and the use of Talking Mats, a facilitative conversation method that supplements interview questions by employing visual cues to clarify options and make them clearer (Nind 2008).

The semi‐structured enabled the first author to be flexible with the questions, which were formulated with more alternatives, as well as additional adjustments that could be made in the interview situation. As supported by Hollomotz (2017), adjusting the depth of questioning to match what the respondent is willing or able to provide improves the quality of the data collected.

The interview questions were mainly open‐ended supplemented with follow‐up questions (see Appendix B). However, such questions might result in situations where people with intellectual disabilities are incapable of answering or provide minimal information resulting in inadequate responses (Coons and Watson 2013; Emerson et al. 2004). To minimise acquiescence and obtain valid responses, it is important to carefully consider the questions asked and the wording used (Prosser and Bromley 2012). Therefore, the first author leveraged insights gained from the established relationship through prior participant observations to tailor the questions. This is in line with other studies that advise researchers to familiarise themselves with the participants before conducting interviews as it can provide the researcher with a clearer understanding of the necessary adaptations and create the optimal conditions for tailoring questions accordingly (Hollomotz 2017; McFarland et al. 2024; Nind 2008). Consistent with the key points provided by this study's expert panel, Emerson et al. (2004) highlighted the importance of questions being brief, straightforward, clear and without complex vocabulary or jargon. The interviewer should speak at a moderate pace, be clear and wait patiently for a response, as responses might take longer than for someone without an intellectual disability. Additionally, rephrasing questions, repeating and summarising responses can be valuable to enhance understanding (Emerson et al. 2004; McFarland et al. 2024).

**FIGURE 3:**
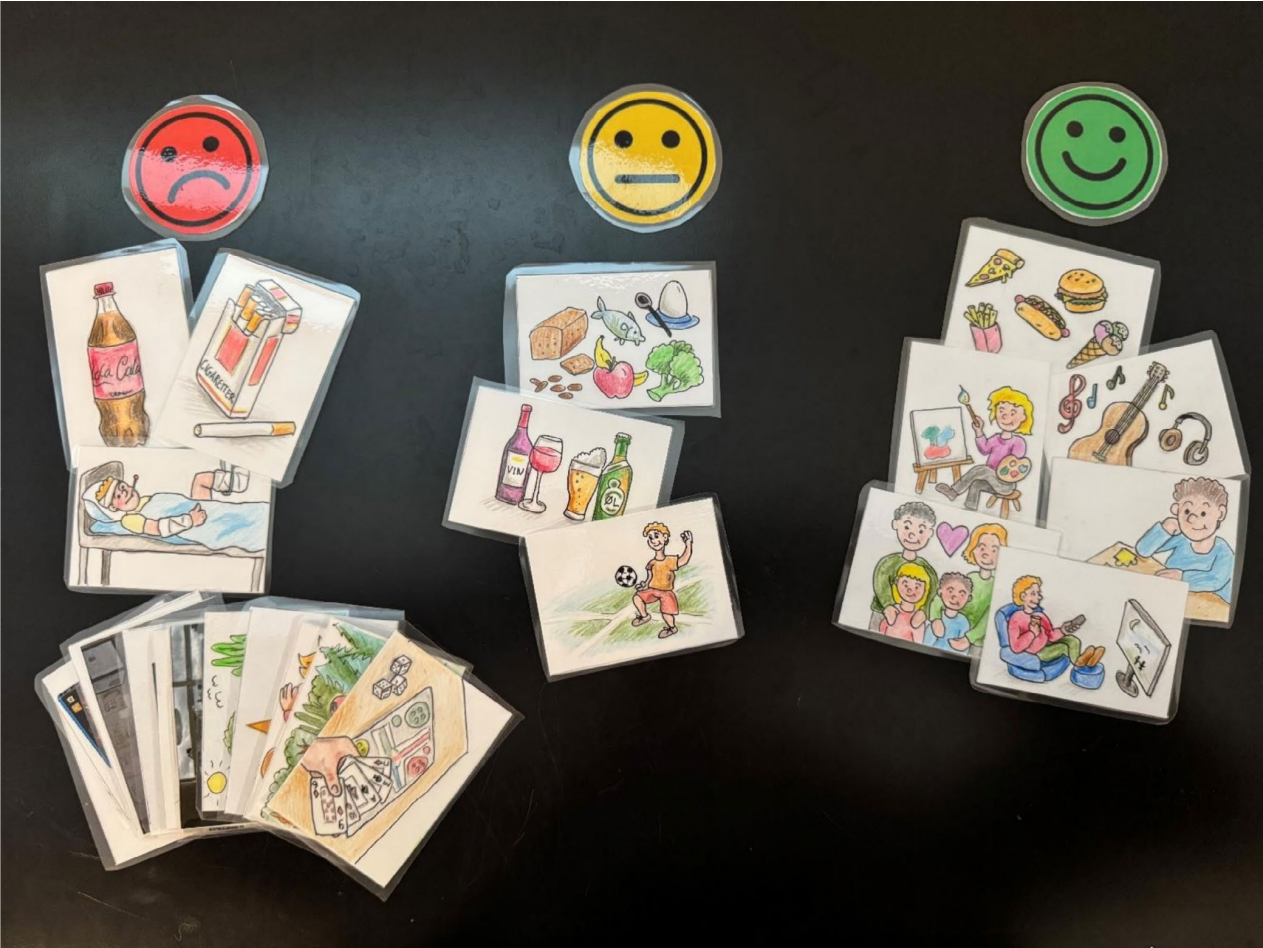
Talking Mats with visual cues.

Alongside interview questions, this study used Talking Mats with visual cues, as a guided conversation method. Providing various opportunities for people with intellectual disabilities to express themselves through multiple modalities can provide structure, complement open‐ended approaches, support the elicitation process and responses without constraining or biasing them, and significantly improve the quality of the information gathered (Lewis et al. 2008; Nind 2008; Prosser and Bromley 2012). The Talking Mats in this study used visual cues to aid comprehension of the interview questions, sustain focus and facilitate participants' self‐expression (Cameron and Murphy 2006; Murphy and Cameron 2008). The visual cues included drawings and pictures, photos taken at the group home and progressively ‘happier’ faces (see Figure 3). Additionally, the first author provided sticky notes, enabling participants to add relevant illustrations during the interview by drawing collaboratively with the first author.

Verbal communication played a key role in our interview approach; however, critical disability studies challenge traditional, verbally dominated interview techniques and their narrow definitions of competence and capacity. Instead, researchers in this field advocate for more inclusive and collaborative approaches to knowledge production. Approaches such as sensory‐dialogue challenge dominant understandings of voice by recognising that communication encompasses embodied expressions, gestures, non‐speech vocalisations and sensory experiences (Gjermestad et al. 2023; Teachman et al. 2018). This aligns with Bakhtinian concepts of dialogism, which position voice as always co‐created rather than individually owned (Bakhtin 1981). Such approaches may benefit both the more traditional interview approaches and the inclusion of people with profound and multiple learning disabilities.

## 6 Balancing Power Dynamics When Involving People With Intellectual Disabilities and Other Relevant Experts

The intention behind assembling a diverse expert panel was to incorporate a range of perspectives, ideally fostering equal and dynamic interactions and contributions. It was also intended to prevent what Srdanovic et al. (2024) refer to as ‘failing ethnographies’, where traditional research methodologies often privilege certain types of researchers, typically those who are ‘unencumbered’ by care responsibilities or disability, while marginalising others. In this study, we faced challenges in creating space for diverse voices and perspectives, especially given the complex power dynamics inherent in the heterogeneous composition of the expert panel. This might have risked marginalising some voices or generating unintended feelings such as sadness or disappointment (Woelders et al. 2015). Navigating these dynamics between researchers inside and outside the academy, each with distinct claims to authority, presents a unique and ongoing challenge within inclusive research (Nind 2017). The expert panel consisted of six members, who were diverse in terms of age, gender and educational background. The panel included both members with and members without intellectual disabilities. To address power dynamics and facilitate equal contributions, the first author initiated the first meeting with an introductory round in which each expert shared their background, research interests and potential contributions. Woelders et al. (2015) emphasised that pursuing normalisation and sameness can hinder progress and obstruct the development of inclusive research. Conversely, embracing diversity and individuality can enrich research outcomes, prompting deeper self‐reflection and challenging the occasionally rigid scientific frameworks. Based on this, the first author attempted to tailor communication to foster the involvement of all members of the expert panel. This included using multiple modalities to accommodate different literacy levels and to support those who were unable to read written text. Consequently, all materials distributed for meetings and meeting minutes were provided both as written documents and as PowerPoint presentations featuring visuals such as illustrations, voice‐overs or video recordings. To assess the impact of the initiatives, the first author conducted a dialogue‐based evaluation of the members' experiences with collaboration and the overall process at its conclusion. The evaluation was conducted both individually via telephone and collectively during an online meeting, ensuring that participants could express themselves freely while also benefiting from each other's reflections. None of the participants reported experiencing power dynamics; instead, they emphasised feeling heard, having the opportunity to contribute and being able to represent their individual experiences.

The initiatives undertaken in this study reflect the recommendations of Woelders et al. (2015), who advocated for ongoing reflexivity among researchers regarding their collaborative practices, the importance of recognising the space given to the unique perspectives of their research partners, and acknowledging their own limitations. In this regard, the concept of ‘failing ethnographies’ offers a particularly productive lens for understanding how critical disability studies may transform research culture. Rather than viewing methodological adaptations as compromises or failures to meet gold standard research criteria, these approaches reframe such adaptations as opportunities to develop more responsive and ethical research practices (Srdanovic et al. 2024).

## 7 Conclusion and Recommendations

Our paper is a contribution to existing research methodologies based on a critical reflection and discussion on the challenges embedded in conducting qualitative research with people with intellectual disabilities. Inclusive research and critical disability studies inspired the analytical lens applied in the discussion on the authors' experiences with conducting an ethnographic study with people with intellectual disabilities and the positioning as inclusive research. The authors recommend the following when conducting research with people with intellectual disabilities:

- Inclusion should be determined on a case‐by‐case basis, ensuring that participants are able to express their willingness to participate.
- To facilitate the inclusion of people with profound intellectual disabilities, the sensory‐dialogical approach offers a promising pathway.
- When obtaining informed consent, it is crucial to allocate sufficient time for participants to fully understand the research and make informed decisions about their participation.
- Supported by the Easy Read format, the expert panel emphasised the importance of balancing information when obtaining informed consent. This information should be delivered through tailored communication, be complemented by illustrations and highlight the right to refuse participation, and researchers should ensure the involvement of a support person when necessary.
- Process consent should be implemented as a supplementary measure to enhance confidence in the participants' genuine desire to participate.
- When conducting participant observations, attention must be given to tailoring note taking based on participants' reactions to the situation, ensuring that it aligns with the specific context, individual preferences and environmental factors.
- Emotional attachments through involvement require the researcher to be reflexive about how potential harm can be managed when exiting the field.
- Potential nervousness or anxiety associated with interviews should be managed through initiatives taken by the researcher before, during and after the interview. It is recommended that the researcher familiarise themself with the participant before an interview to create a relaxed atmosphere and tailor the communication accordingly
- For researchers with limited experience in interviewing people with intellectual disabilities, involvement with an expert panel can offer essential expertise and insights to inform the development of interview guides and questions
- Providing multiple ways for participants to express themselves can enhance their ability to communicate. This might include tools such as Talking Mats and visual cues. For further development of verbally dominated interview approaches, the sensory‐dialogical approach acknowledges multiple modes of expression.
- Potential power dynamics within an expert panel consisting of members both inside and outside academia require ongoing reflexivity from the researcher. This should include strategies to acknowledge diversity and approaches that create space for different perspectives, such as highlighting everyone's contributions and tailoring communication through multiple modalities. Furthermore, conducting an evaluation of members' experiences of the process and collaboration upon its conclusion may constitute an effective approach to uncover potential power dynamics with implications for future involvement.

While significant progress has been made in developing inclusive research methodologies, substantial challenges remain. Future methodological developments may build on our contribution and the sensory‐dialogical approach proposed by Gjermestad et al. (2023) which offers one promising direction for future development, particularly in its recognition that inclusion requires fundamental rethinking of how knowledge is produced and validated. Such development requires continued attention to training and support for researchers. As this paper reveals, conducting qualitative research with people with intellectual disabilities demands specific competencies around relationship‐building, ethical practice and communication that might not be adequately addressed in traditional research training programmes. The ultimate goal of inclusive research is not merely to include people with intellectual disabilities within existing research frameworks, but to transform those frameworks to create opportunities to develop more responsive and ethical research practices (Srdanovic et al. 2024). This transformation necessitates ongoing dialogue within the research community that actively involves people with intellectual disabilities, continuously innovative methodology and institutional changes that support more diverse forms of knowledge production. As the field continues to develop, the challenge remains to ensure that inclusive research truly serves the interests of people with intellectual disabilities while contributing to broader social and political struggles for disability justice.

## Author Contributions

K.N.F., S.L.V., M.G., P.R. and H.H.‐T. conceptualised and designed the work. K.N.F. was responsible for data acquisition, which forms the basis of the discussion presented in this paper. K.N.F. drafted the initial manuscript, while S.L.V., T.F., M.G., P.R. and H.H.‐T. contributed to data analysis, interpretation, manuscript development and provided critical revisions. All authors have read and approved the final version of the manuscript.

## Funding

The study received support from the Department of Nursing, University College of Northern Denmark; Department of Clinical Medicine, Aalborg University; Aalborg Municipality.

## Disclosure

The authors have nothing to report.

## Ethics Statement

The study complied with the General Data Protection Regulation (GDPR) and was registered at Aalborg University (AAU) under ID number 2022‐068‐02569. Ethical approval was granted by the North Denmark Region Committee on Health Research Ethics (journal number: 2022‐000764). Furthermore, the research adhered to the principles outlined in The Danish Code of Conduct for Research Integrity throughout its design and execution.

## Consent

All participants provided both verbal and written informed consent after receiving information about the study in verbal and written form from the researcher. Participants were assured anonymity, confidentiality and voluntary participation, and pseudonyms were assigned. Consent was obtained in the physical presence of the first author and a support worker to foster a safe environment and ensure communication in an adapted language. In cases where a participant expressed willingness to participate but had a legal guardian, the guardian was also required to provide written consent.

## Conflicts of Interest

The authors declare no conflicts of interest.

## Data Availability

The interview guide is available from the first author upon request. Due to privacy and ethical concerns, the data supporting the study's findings are not publicly accessible.

## Appendix A Poster. Translated From Danish Into English

Project: Decisions About Health.

Hello, my name is

I would like to write a book about you, who live in this group home, and how you make decisions about your health together with the staff. The book will also include experiences from people with intellectual disabilities living in other group homes.

I am not a new support worker, and I will not be living here at the group home.

I will be visiting the group home about 1–3 days a week for approximately 3 months. During my visits, I would like to spend time with you while you cook, eat together, watch TV and in many other situations.

While I am with you, I will write down some notes on paper that I can use for my book. Sometimes, I may also ask if I can take a photo of what you are doing, such as the food you are preparing in the kitchen.

At some point, I might ask if you would like to be interviewed. An interview is a conversation between you and me, where you can tell me about your daily life and how you make decisions about your health together with the staff.

In my book, you will remain anonymous. This means you will be given a different name in the book, so no one will know it was you who said it. For example, if you tell me something you don't want others to know, it won't be identifiable as coming from you.

If you don't want to talk to me or don't want me to observe what you are doing, just let me, a staff member or someone else know. That's completely okay.

Best regards

Contact Information

## Appendix B Examples of Open‐Ended Interview Questions and Follow‐Up Questions

**Table:**

Interview‐questions	Follow‐up questions
How do you understand health?	If health is more than that, what could it be?
Talking Mats and visual cues are put into use Try using some of the visual cues	What is on it? Why did you choose it? Which smiley would you like to put it on, and why? Does it have anything to do with your own life, or someone else's?
